# Drivers of men’s use of intimate partner violence in conflict-affected settings: learnings from the Democratic Republic of Congo

**DOI:** 10.1186/s13031-023-00562-5

**Published:** 2024-01-22

**Authors:** Christine Bourey, Rashelle J. Musci, Judith K. Bass, Nancy Glass, Amani Matabaro, Jocelyn T. D. Kelly

**Affiliations:** 1 Department of Epidemiology, Columbia University Mailman School of Public Health, New York, NY USA; 2 Johns Hopkins University School of Nursing, Baltimore, MD USA; 3 Action Kivu, Bukavu, Democratic Republic of Congo; 4 Harvard Humanitarian Initiative, Harvard University, Boston, MA USA; 5 Brigham and Women’s Hospital, Boston, MA USA

**Keywords:** Intimate partner Violence (IPV), Perpetration, Armed conflict, Democratic Republic of Congo

## Abstract

**Background:**

Intimate partner violence against women (IPVAW) is prevalent in conflict-affected settings. Yet, there is limited knowledge about the risk factors that influence men’s use of IPVAW in conflict-affected settings. This paper adopts a transdisciplinary perspective to understand how experiences hypothesized to increase men’s use of IPVAW relate to each other and to men’s use of IPVAW. The findings may help researchers and interventionists to better select and target interventions for IPVAW in conflict-affected settings.

**Methods:**

We used baseline data from the Tushinde Ujeuri project in the Democratic Republic of Congo. Men with at least partial data for the variables of interest were included in the analysis (n = 2080). We estimated a structural equation model that explored how five constructs – interpersonal violence, mental health, socioeconomic adversity, gender inequitable attitudes, and conflict violence – influenced men’s self-reported past-year use of physical and/or sexual IPVAW.

**Results:**

The model had acceptable fit (χ^2^ = 1576.574, p = 0.000; RMSEA = 0.041; CLI = 0.882; SRMR = 0.055). There was a statistically significant path from interpersonal violence to IPVAW (β = 0.875; OR = 2.40). Interpersonal violence also was linked to gender inequitable attitudes (β = 0.364), which were linked to increased use of IPVAW (β = 0.180; OR = 1.20). Moreover, interpersonal violence was linked to trauma symptoms (β = 0.331), which were linked to increased use of IPVAW (β = 0.238; OR = 1.27). Use of IPVAW decreased as conflict exposures increased (β=-0.036; OR = 0.96), and there was no path from socioeconomic adversity to IPVAW.

**Conclusions:**

Our findings suggest interpersonal violence exposures, trauma symptoms, and gender inequitable attitudes are all risk factors for the use of IPVAW in a conflict-affected setting. While continuing to focus on gender inequitable attitudes and norms, interventionists should also consider addressing men’s experiences of victimization and mental wellbeing. Doing so can help to improve trauma symptoms and may hold promise to reduce IPVAW in conflict-affected settings.

**Supplementary Information:**

The online version contains supplementary material available at 10.1186/s13031-023-00562-5.

## Background

Intimate partner violence (IPV) against women (IPVAW) is a critical public health issue in conflict-affected settings. Defined as behavior within an intimate relationship that threatens or causes physical, psychological, or sexual harm to women [1], IPVAW perpetrated by men is the most common form of violence against women in these settings [2]. Emerging evidence suggests exposure to conflict violence and living in conflict-affected communities may increase women’s risk for experiencing IPVAW [3–7], and the confluence of multiple adversities and insufficient protective factors may compound the effect of IPVAW on women’s physical and mental health [8, 9].

Understanding risk factors for men’s use of IPVAW in conflict-affected settings is critical for understanding why this violence occurs and for informing interventions to address it. The mechanisms driving men’s use of IPVAW in conflict-affected settings, however, are not well understood, potentially reflecting the relative paucity of research on men’s use of violence compared to women’s experience of violence [2]. Perhaps consequently, few IPVAW interventions engaging men have proven efficacious. Among ten studies engaging men to reduce IPVAW in conflict and post-conflict settings [10–19], three have shown a statistically significant reduction in any type of IPVAW when compared to a control group: community-based dialogue groups [19], gender dialogue groups added to group savings [10], and trauma-informed psychotherapy [17].

Current evidence has identified multiple risk factor targets for prevention of IPVAW in conflict-affected settings [20]. Some risk factors are common to non-conflict settings, such as age [21–24], polygamous marriage [24], income [24], childhood abuse [24], witnessing intra-parental violence [22, 23], permissive attitudes toward IPV [22], and psychological symptoms and substance misuse [23–26]. There also are risk factors more specific to conflict settings including exposure to torture [27], history of migration or displacement [24], and exposure to conflict violence [3, 4, 6, 28, 29].

Few studies model relationships among the different experiences and characteristics that impact men’s use of IPVAW in conflict-affected settings [27]. Understanding relationships among experiences and characteristics related to men’s use of IPVAW can improve interventions by helping to identify if an experience or characteristic is more proximal or distal, exerts an independent effect or functions through a mediator, and what other things will be affected by changing the experience or characteristics, thereby identifying potential intervention mechanisms, synergies, or untoward effects. Among studies that consider relationships among experiences and characteristics, none explicitly adopts a transdisciplinary perspective, investigating the multiple ways that exposures affect IPVAW in conflict-affected settings (i.e., they typically focus on experiences and characteristics from a single domain while IPVAW requires more complex modeling). In this paper, we present a model that examined how multiple constructs (i.e., experiences represented by one or more variables), drawn from different domains, interrelate to increase men’s use of IPVAW in a conflict-affected area of the Democratic Republic of Congo.

Different health and social science disciplines (e.g., public health, clinical psychology, econometrics) have developed hypotheses that focus on different domains (e.g., attitudes, behaviors, and norms; experiences of traumatic stress; experiences of poverty) to explain men’s use of violence. We focused on five constructs from different domains that may impact IPVAW in conflict-affected settings: men’s experiences of interpersonal violence, mental health, socioeconomic adversity, gender inequitable attitudes, and conflict violence. Briefly, interpersonal violence, such as child abuse, can prime intergenerational cycles of violence [30], exacerbate socioeconomic adversity [31], contribute to mental health symptoms [32], and heighten gender inequitable attitudes (e.g., where witnessing interparental IPV teaches gender inequitable attitudes like the belief that there are times when a woman deserves to be beaten) [33]. Conflict-related experiences also can produce mental health symptoms that compromise emotional regulation, lead to attribution of negative partner intent, or create additional stress in relationships, increasing risk for IPVAW [34, 35]. Armed conflict can exacerbate socioeconomic adversity [36], and economic stressors can overwhelm available resources, thus generating a crisis in families leading to IPVAW [37]. Gender inequitable attitudes, behaviors, and norms [38] can result in unequal power that increases risk for the use of IPVAW [39, 40]. Exposure to conflict violence can normalize violence [41], such that aggression may be seen as an effective way to resolve conflict [42].

This paper undertook structural equation modeling to understand how these five different constructs may contribute to IPVAW, and how they may relate to each other. To date, nearly all studies have used multivariable logistic regression, which does not account for complex relationships, such as the presence of mediating variables. By considering multiple domains that are the focus of different disciplines (i.e., adopting a transdisciplinary perspective) and using this advanced statistical approach, we can better understand the relationship of these constructs to IPVAW in a conflict-affected setting, helping researchers and interventionists to better select and target interventions for IPVAW in conflict-affected settings.

## Methods

### Design and setting

DRC is a large central African country roughly equivalent in size to western Europe. Much of the country has experienced longstanding armed conflict [43] including periods of intense conflict during the first (1996–1997) and second Congo Wars (1998–2003). Although the intensity of armed conflict has vacillated since the official end of the armed conflict, militias have continued to proliferate [44]. Gender-based violence also is prevalent in DRC. 57% of ever-married women of reproductive age (15–49 years old) reported lifetime IPVAW, as defined by physical, sexual, and/or emotional violence from their husband/partner. Of these women, 77% reported IPVAW within the past year [45]. This is compared to less than one quarter of women who reported non-partner sexual violence [29, 46].

This secondary analysis used baseline data from the Tushinde Ujeuri project funded by the United States Agency for International Development (USAID, DRG Learning, Evaluation, and Research I award). Additional information about the study is available elsewhere [47]. In brief, the Tushinde Ujeuri project aimed to help communities prevent and respond to gender-based violence in eastern DRC. At baseline, villages were selected purposively based on eligibility for project implementation. To select households for baseline interviews, field teams used a random walk methodology to select approximately 20 households for each of 224 villages. One woman or man from each household was selected at random from a list of usual members of the household. Male interviewers administered the survey to men, and female interviewers administered the survey to women. In total, baseline data was collected from 2108 men, 2114 women, and 1 nonbinary person. This study focused on the male sample, using 2080 men who had at least partial data on variables of interest for this analysis. The sample was not restricted by relationship status because it was not adequately measured or assessed (33% missingness). Moreover, 22% of men who identified as “single, never married” reported using IPVAW, suggesting restriction to currently partnered/married men would miss an important demographic, even if restriction were made possible through imputation.

### Measures

Several individual-level questions relevant to IPVAW were included in the survey. Among these were: (1) demographics, (2) exposure to interpersonal violence such as witnessing interparental IPV, (3) mental health symptoms, (4) socioeconomic experiences, (5) gender inequitable attitudes and social norms, (6) conflict exposures such as experiences of human insecurity, and (7) use of IPVAW. We selected variables related to the five constructs outlined in the introduction: interpersonal violence, mental health, socioeconomic adversity, gender inequitable attitudes, and conflict violence.

#### Constructs hypothesized to contribute to IPVAW

We operationalized each construct in one of two ways: (1) by using a previously developed scale or index or (2) by examining all measured variables related to the construct and reducing to a core set. Scales or indices were available to organize measured variables for mental health symptoms and gender inequitable attitudes. For three constructs (interpersonal violence, socioeconomic adversity, and conflict violence), we used exploratory factor analysis (EFA) to identify variables that could represent the construct (see below for additional description of EFA).

##### Interpersonal violence

For interpersonal violence, four variables were available: familial physical child abuse (never, sometimes, often), witnessing parental IPV (no/yes), lifetime sexual violence victimization (no/yes), and adult physical violence victimization (no/yes).

##### Mental health symptoms

Mental health symptoms, hereafter referred to as trauma symptoms, were measured by the Harvard Trauma Questionnaire. The scale was selected, adapted, and tested in-country using an approach described elsewhere [47]. The Harvard Trauma Questionnaire included 16 items measuring posttraumatic stress disorder (PTSD) symptoms (Cronbach’s ⍺ = 0.88). For each item, respondents were asked to describe how much the symptom had bothered them in the last month: not at all (0), a little (1), moderately (2), or a lot (3). The average of the 16 items was used (0–3).

##### Socioeconomic adversity

For socioeconomic adversity, we investigated five variables: poverty (210,000+, 150,000-209,999, 60,000-149,999, 30,000–59,999, 10,000–29,999, 1-9999, 0 Congolese franc), standard of living (count of 11 household assets such as a radio or electricity; treated as a continuous variable), unemployment (no/yes), perceived household living conditions compared to neighboring households in the village (much worse, somewhat worse, about the same, somewhat better, much better), and perceived village living conditions compared to neighboring villages (much worse, somewhat worse, about the same, somewhat better, much better).

##### Gender inequitable attitudes

Gender inequitable attitudes were measured through an index of 14 statements to which respondents indicated their degree of agreement. For example, respondents were presented with the statement, “a woman should obey her husband in all things,” and asked to respond whether they strongly disagree (-2), somewhat disagree (-1), neither agree nor disagree (0), somewhat agree (1), or strongly agree (2). The average of these 14 items was used (-2–2).

##### Conflict volence

For conflict violence, we investigated eight variables: adult physical violence victimization (no/yes), lifetime sexual violence victimization (no/yes), witnessing conflict violence (count of six events such as witnessing harassment and torture; treated as a continuous variable), experiencing conflict violence (count of 10 events such as experiencing looting or theft of assets/supplies; treated as a continuous variable), perceived safety of the village (very unsafe, unsafe, neither safe nor unsafe, safe, very safe), human insecurity index (average of seven questions for which respondents selected never, sometimes, often), current or lifetime displacement as a result of armed conflict (no/yes), and time to reach the nearest drivable road (minutes). We included the last variable as a proxy for community violence exposure; past findings suggested relationships between road density and exposure to conflict violence in Africa [51].

#### Outcome

The primary outcome was past-year use of physical and/or sexual IPVAW. We selected this timeframe to reduce temporal bias. Men were asked: “In your relationship with your (last) wife/girlfriend/partner do (did) the following happen never, sometimes, or frequently: You hit, slapped, kicked, or did anything else to hurt your current or previous wife or girlfriend physically? You forced your current or previous wife or girlfriend to have sex with you or perform another sexual act when she did not want to?” After each question, men who answered sometimes or frequently were asked if they had done any of these things in the past 12 months. Men who answered yes to either physical or sexual IPVAW use within the past 12 months were coded as using past-year IPVAW.

### Statistical analyses

#### Exploratory factor analysis and descriptive statistics

Where there was not an available scale or index to represent a construct (i.e., interpersonal violence, socioeconomic adversity, and conflict violence), we performed EFA using the WLSMV estimator, with adjustment for clustering at the village level. As applied in this study, EFA is a statistical technique that facilitates iterative examination of a group of variables and enables removal of variables until there is a cluster of intercorrelated variables that load on a single (latent) factor. We privileged a single factor loading so that each group of variables would represent a single construct. We used the resulting group of variables (i.e., measurement model) to represent the construct in the structural equation model (SEM). EFA was chosen over confirmatory factor analysis because there was not significant theory and empirical findings to test hypothesized groups of variables; rather, we aimed to uncover relationships present in the data.

We then calculated descriptive statistics for variables representing interpersonal violence, trauma symptoms, socioeconomic adversity, gender inequitable attitudes, conflict violence, and the outcome.

#### Structural equation model

We used a SEM approach to explore how interpersonal violence, trauma symptoms, socioeconomic adversity, gender inequitable attitudes, and conflict violence might influence IPVAW. SEM is a statistical technique in which one or more groups of variables representing a latent construct (i.e., measurement model) predict one or more outcomes (i.e., structural model). The model commonly is drawn as a series of ovals (latent variables) and rectangles (observed variables) connected by directional arrows. Each arrow specifies a hypothesized causal relationship, which is one assumption of the statistical model [48]. To define these casual relationships, we drew on the empirical literature and expert knowledge of the field.

##### Primary hypotheses

We hypothesized that interpersonal violence, trauma symptoms, socioeconomic adversity, gender inequitable attitudes, and conflict violence would directly affect IPVAW, as described in the introduction.

##### Secondary hypotheses

**Fig. 1:**
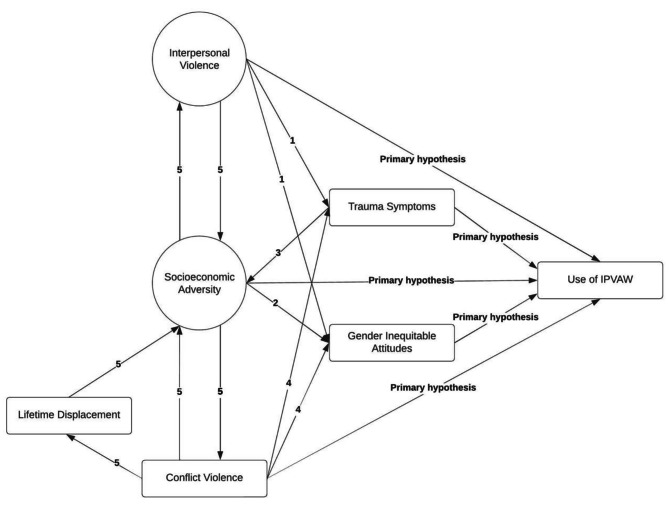
Hypothesized relationships among five constructs and men’s use of IPVAW

We also hypothesized relationships among constructs. These are detailed in Fig. 1, which presents the SEM without the measurement models. We hypothesized:

1. interpersonal violence exposure would lead to trauma symptoms and gender inequitable attitudes, the latter because experiences such as child abuse and witnessing interparental IPV can normalize familial violence;
2. socioeconomic adversity would exacerbate gender inequitable attitudes: for example, when unemployment threatens a man’s fulfillment of traditional gender roles leading him to reclaim his masculine identity through increased justification and use of IPVAW. By contrast, socioeconomic adversity cannot cause trauma symptoms (as defined by the Diagnostic and Statistical Manual 5);
3. trauma symptoms would function in the opposite direction: exacerbating socioeconomic adversities where symptoms result in functional impairment;
4. conflict violence would increase trauma symptoms and gender inequitable attitudes, the latter when conflict violence causes multiple types of violence including IPVAW to be normalized and justified;
5. reciprocal relationships would exist between (a) interpersonal violence and socioeconomic adversity and (b) conflict violence and socioeconomic adversity, and displacement would partially mediate the relationship between conflict violence and socioeconomic adversity.

After estimating the model, we examined fit statistics (χ^2^, RMSEA, CLI, and SRMR) and modification indices. We centered theory and past empirical findings when selecting model adjustments. The final model balanced model fit and parsimony.

##### Sensitivity and additional analyses

We conducted two sets of sensitivity analyses. One controlled for age. The other estimated the main SEM on a restricted sample that excluded anyone who reported (a) adult physical violence and experience of being physically beaten by an armed group or (b) lifetime sexual violence and experience of sexual abuse by an armed group. This sensitivity analysis was undertaken because we were unable to fully distinguish between interpersonal violence and conflict violence exposures for men who reported both exposures (i.e., men who reported adult physical violence may have endorsed this item due to their experience of physical violence within conflict), leading to possible misclassification bias. We also conducted an analysis using depression/anxiety symptoms in place of trauma symptoms. We have chosen to present these findings in an online supplement to streamline the analysis. We privilege the analysis with trauma symptoms because these symptoms have a stronger theoretical and empirical basis for a causal effect.

Descriptive statistics were calculated in Stata 14. All EFA and SEM analyses were conducted in MPlus 8. Full information maximum likelihood estimation was used in EFA and SEM models to use all available data. Statistical significance was evaluated at the conventional p < 0.05 level. The Tushinde Ujeuri baseline and endline surveys were approved by the NORC Institutional Review Board.

## Results

Table 1 describes the 2080 male study participants. Overall, the sample was highly trauma exposed. Most men reported physical childhood abuse (83.8%) and witnessing interparental IPV (51.0%). Approximately one-third reported adult physical violence (33.1%). Lifetime sexual violence was least commonly reported (8.4%). On average, men witnessed or experienced 3.8 conflict-related events, and more than half (53.8%) of the sample reported experiencing displacement in their lifetimes.

**Table 1:** Descriptive statistics for variables included in the structural equation model

	n	% (n) or mean (sd)
Physical childhood abuse, % (n)	2012	
Never		16.3 (328)
Sometimes		58.7 (1180)
Often		25.1 (504)
Witnessed parental IPV, % (n)		
Never	1908	49.1 (936)
Sometimes		39.1 (745)
Often		11.9 (227)
Lifetime sexual violence victimization, % (n)	2108	8.4 (176)
Adult physical violence victimization, % (n)	2108	33.1 (698)
Household income/poverty		
210,000 CDF and higher	2106	10.0 (210)
150,000-209,999 CDF		9.7 (204)
60,000-149,999 CDF		33.2 (699)
30,000–59,999 CDF		23.7 (500)
10,000–29,999 CDF		10.7 (226)
1–9,999 CDF		1.1 (24)
0 CDF		11.5 (243)
Unemployment, % (n)	2108	67.1 (1414)
Perceived household disadvantage, % (n)		
Much worse	2088	7.7 (161)
Somewhat worse		29.6 (618)
About the same		20.6 (430)
Somewhat better		38.8 (809)
Much better		3.4 (70)
Perceived village disadvantage, % (n)		
Much worse	2055	4.5 (92)
Somewhat worse		21.8 (448)
About the same		31.5 (648)
Somewhat better		37.8 (776)
Much better		4.4 (91)
Lifetime displacement, % (n)	2108	53.8 (1135)
Exposure to conflict violence, mean (sd)	2108	3.8 (4.2)
Trauma symptoms, mean (sd)	1885	0.7 (0.6)
Gender inequitable attitudes, mean (sd)	1965	0.1 (0.7)
Past-year use of IPVAW, % (n)	2046	16.2 (332)

Regarding socioeconomic disadvantage, although there was a broad range of income, 11.5% of men reported no household income, and 67.1% of men reported unemployment. Perception of relative household and community disadvantage was clustered in the middle, with most men having responded somewhat worse (household: 29.6%, community: 21.8%), about the same (household: 20.6%, community: 31.5%), or somewhat better (household: 38.8%, community: 37.8%) than neighboring households or communities.

On average, there was slight agreement with gender inequitable attitudes (0.12; scale range − 2–2). Men reported none to little trauma symptoms on average (0.69; scale range 0–3). 16% (16.2%) of men reported past-year use of physical and/or sexual IPV.

### Exploratory factor analysis

We performed EFA to select variables to represent three constructs: interpersonal violence, socioeconomic adversity, and conflict violence. In the EFA for interpersonal violence exposure, all variables were retained in a one-factor model (χ^2^ = 5.111, p = 0.078; RMSEA = 0.027; CLI = 0.991; SRMR = 0.039). For socioeconomic adversity, the final solution was a one-factor model with all variables except standard of living (χ^2^ = 7.705, p = 0.021; RMSEA = 0.037; CLI = 0.996; SRMR = 0.027). We found a two-factor solution with reasonable fit for conflict violence exposure. This solution, however, had many cross-loadings and few/low loadings on the second factor, suggesting the absence of two factors measured by distinct variables. We, therefore, decided to use a measured variable that was a combination of witnessing and experiencing conflict violence (0–16 events; treated as a continuous variable). We also included lifetime displacement as a separate variable.

### Structural equation model

**Fig. 2:**
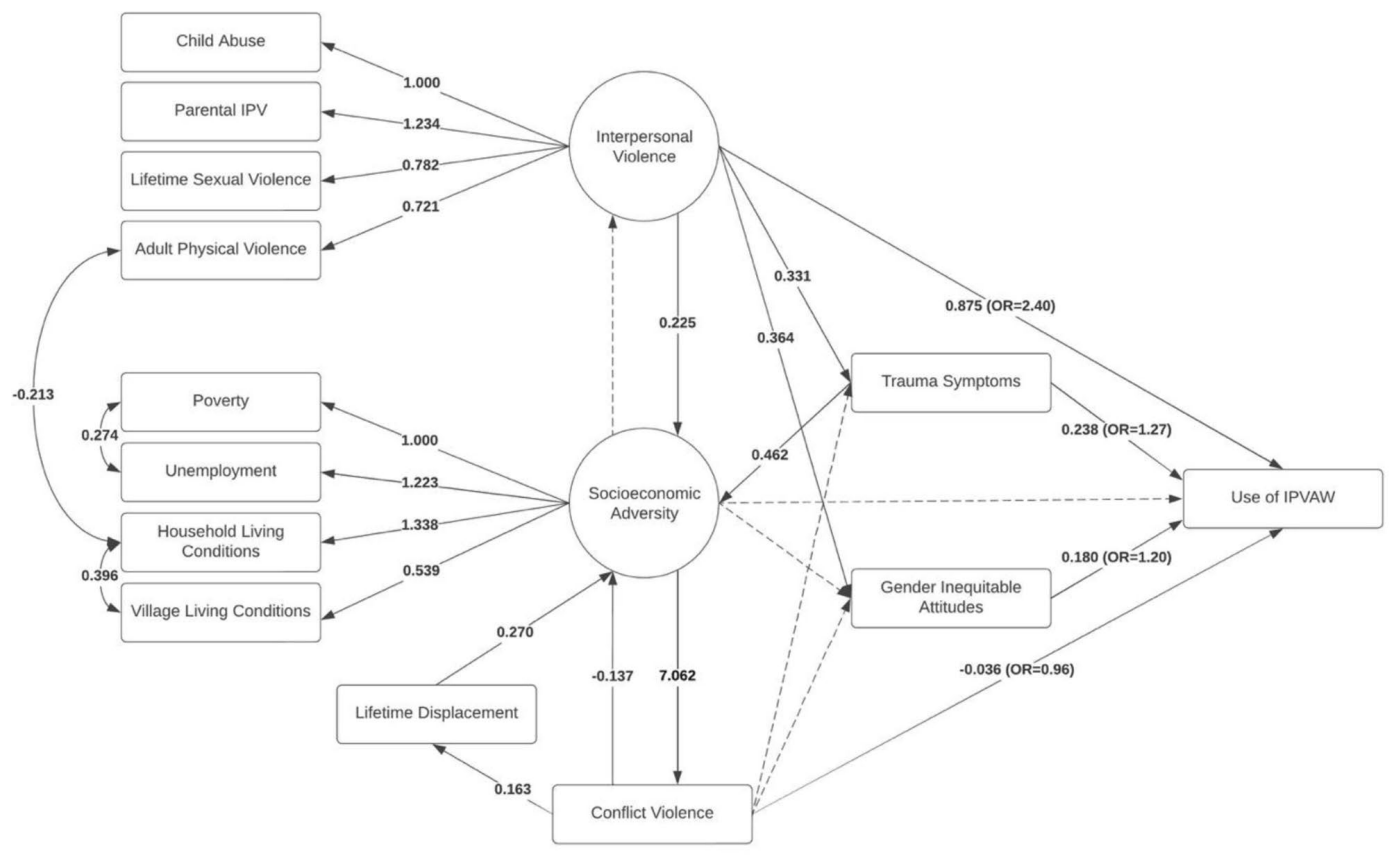
Structural equation model of men’s use of IPVAW (n = 2080)

The SEM had acceptable fit (χ^2^ = 1576.574, p = 0.000; RMSEA = 0.041; CLI = 0.882; SRMR = 0.055) (Fig. 2). Regarding primary hypotheses: Interpersonal violence was linked directly to IPVAW (β = 0.875; OR = 2.40). Interpersonal violence also was linked to trauma symptoms (β = 0.331), which were linked to use of IPVAW (β = 0.238; OR = 1.27). There was no path, however, from socioeconomic adversity to use of IPVAW. There was a path from interpersonal violence to gender inequitable attitudes (β = 0.364) to increased use of IPVAW (β = 0.180; OR = 1.20). Whereas there was a direct relationship between conflict exposure and use of IPVAW, use of IPVAW decreased as conflict exposures increased (β=-0.036; OR = 0.96).

### Sensitivity analyses

In the sensitivity analyses controlling for age (Supplement, Fig. 1), patterns of results did not change. In the sensitivity analyses considering potential overlap in physical or sexual violence experiences and conflict violence exposures (n = 1888) (Supplement, Fig. 2), the primary relationships for the five constructs and IPVAW did not change.

## Discussion

Our study is among the first to empirically assess relationships among constructs, each of which speaks to a different hypothesis of drivers for the use of IPVAW in a conflict-affected setting. If the model’s assumptions are correct, including the direction of causality, men’s experiences of interpersonal violence lead to increased use of IPVAW. Moreover, men’s experiences of interpersonal violence lead to trauma symptoms and gender inequitable attitudes, which lead to IPVAW use. By contrast, men’s experiences of socioeconomic adversity do not contribute directly or indirectly to the use of IPVAW, and men’s experiences of conflict violence are inversely related to the use of IPVAW.

### Interpersonal violence

The link between select types of interpersonal violence exposure (e.g., child abuse) and men’s use of IPVAW is well established in the current literature [3, 4, 29, 30, 49, 50], although less well documented in literature focused on conflict-affected settings. Scholars have explained this connection in varied ways, including through social learning theories that emphasize how direct experiences of violence contribute to the internalization of complex scripts condoning violence [51]. Through exposure to violence, violence can be normalized [41], and aggression may be seen as an effective way to resolve conflict [42]. When these internalized scripts include patriarchal attitudes justifying IPVAW (e.g., when a boy witnesses his father use IPVAW), the relationship between interpersonal violence and men’s use of IPVAW could be mediated through gender inequitable attitudes, consistent with this SEM.

Interventions focused on the relationship between men’s experiences of interpersonal violence and men’s use of IPVAW in conflict-affected settings are relatively rare. One recent exception is Safe at Home, a discussion group intervention in DRC, that explicitly recognized shared structural drivers for IPV and harsh parenting [19], thereby addressing multiple interpersonal violence exposures (e.g., child abuse and witnessing IPVAW) that could contribute to IPVAW use in the present and subsequent generations.

### Trauma symptoms

Our finding of a significant relationship between trauma symptoms and the use of IPVAW adds to a large body of research that demonstrates a relationship between PTSD symptoms or diagnosis and IPV [35, 49, 50]. Most data, however, are limited to high-income countries [49]. Among studies in conflict and post-conflict settings, our findings agree with studies from Liberia, where posttraumatic stress symptoms were associated with the use of IPV after controlling for exposure to conflict events [15], and DRC, where treatment for posttraumatic stress reduced violent behavior [27]. They diverge from a study from Uganda, in which posttraumatic stress symptom severity was not associated with IPVAW [16]. It is possible that multiple contextual factors shape the relationship between posttraumatic stress symptoms and IPVAW. For example, in northern Uganda, women with reexperiencing symptoms had less IPV risk when their husbands also had posttraumatic stress symptoms due to reduced stigmatization of women by their male partners [16]; thus, male posttraumatic stress symptoms were protective in some cases.

Mental health interventions have been underutilized as a tool to reduce the use of IPVAW in conflict-affected settings. In combination with the current literature, our findings suggest trauma-focused mental health interventions could be integrated and tested as part of IPVAW interventions in conflict-affected settings. For example, the Common Elements Treatment Approach (CETA), a transdiagnostic psychotherapeutic intervention, has been used successfully to reduce IPV in a low resource setting [51] and may represent one model that can be combined with current gender equity-focused interventions. Likewise, narrative exposure therapy was proven to reduce violent behavior among male former combatants with PTSD in DRC [27] but may have applicability to the general population.

### Gender inequitable attitudes

Men’s gender inequitable attitudes have been consistently linked to IPVAW [52], and researchers have suggested this link may be amplified in conflict-affected settings [53]. One line of reasoning suggests that, as conflict-affected communities experience changes around social roles that challenge gender inequity (e.g., women working outside the home), men may use IPVAW to maintain power in their relationship, home, and larger community [54].

Dedicated attention to gender norms and attitudes in IPV interventions in conflict-affected settings is critical. Although interventions focusing on gender inequitable attitudes and related social norms have shown mixed effects in controlled studies, some studies have observed robust effects [19] or changes in factors that may precede IPV, including changes in attitudes toward violence 12. Our findings suggest targeting gender inequitable attitudes alongside mental health may be a useful strategy. Aligned with this recommendation, Living Peace in DRC used psychotherapeutic groups to address men’s trauma experiences and gender inequities [55]. Evaluation findings have been positive [18], although a quantitative impact assessment was not available at the time of writing. We emphasize the combination of gender inequitable attitudes and mental health here because interpersonal violence exposures were heterogeneous in terms of timing and setting, suggesting more research is needed on which exposures drive the relationship between interpersonal violence and IPVAW prior to programmatic recommendations.

### Socioeconomic adversity and conflict violence

No association between men’s reported experience of socioeconomic adversity and IPVAW was found, and a statistically significant association was found between increased conflict violence and decreased IPVAW. The latter finding differs from at least one previous study that linked exposure to war-related events with the use of IPVAW by men [15]. It is possible, however, that trauma symptoms explain the association between exposure to conflict-related events and IPVAW. Rees and colleagues [18] found that mental disturbance (including symptoms of PTSD, depression/anxiety, and alcohol abuse) mediated the relationship between experiences of torture and IPVAW in Timor-Leste [18]. If the posited direction of effect is correct for these exposures, microfinance, cash transfers, and similar interventions targeting men may be less useful in this setting. Likewise, focusing on conflict violence exposures may be less fruitful for IPVAW than addressing interpersonal violence exposures: for example, when identifying higher-risk men for selective prevention interventions.

### Strengths and limitations

Our study has several strengths and limitations. To our knowledge, this is the first study to simultaneously explore multiple different disciplinary perspectives on the use of IPVAW in a conflict-affected setting. In so doing, it extends a literature dominated by univariable and multivariable regression models exploring variables correlated with women’s experiences of IPVAW and generates actionable results relevant to public health and humanitarian practice.

Among limitations, our measure of the use of IPVAW captures men’s self-report. Men who report using IPVAW may differ systematically from men who use, but do not report, IPVAW. The direction of bias is unclear as the characteristics of men who use IPVAW but do not report its use are unknown in this setting. It is possible, however, that any such misclassification attenuates the magnitude of our findings. For example, if there is a relationship between gender inequitable attitudes and IPVAW, findings would be attenuated if men demonstrating this relationship were incorrected classified as not using IPVAW. Additionally, our research only included variables measured at the individual level, whereas IPVAW is affected by experiences across the social ecology [56]. Furthermore, no data on alcohol use were available. This is important as alcohol may, at least partly, mediate the relationship between trauma symptoms and use of IPVAW, suggesting an additional focus for intervention. Future studies should attend to the potentially complex interaction between alcohol use and trauma symptoms to guide intervention components. Given multiple exposures were specific to childhood (i.e., witnessing IPV between parents and child abuse), our findings underscore the possibility that addressing violence against children may be a key IPVAW prevention strategy in this setting. More research is needed, however, to disentangle the effects of childhood and adult or lifetime exposures on men’s use of IPVAW in conflict-affected settings.

SEM posits directional relationships, and we employed cross-sectional survey data. We therefore made assumptions about the direction of effect based on theory and empirical literature. It is possible, however, that some relationships could be bidirectional (suggesting endogeneity or simultaneity bias). For example, we modeled the impact of gender inequitable attitudes on the use of IPVAW; the use of IPVAW also may heighten and entrench gender inequitable attitudes. We were unable to model bidirectionality in all cases due to the statistical complexity of the resulting model. Although reverse causality also is possible, this is less likely given the variables used in the model have strong empirical or theoretical bases for influencing the use of IPVAW.

## Conclusions

Continuing to address women’s mental health needs and promote women’s advancement is imperative. Simultaneously, men increasingly are being engaged in IPVAW interventions in conflict-affected settings with mixed efficacy. In this paper, we sought to understand why IPVAW occurs to inform interventions engaging men to address it in conflict-affected settings. By using EFA, we better represented the complex constructs that may influence men’s use of IPVAW. Through a SEM approach, we modeled complicated relationships among these constructs, simultaneously considering domains emphasized by different disciplines and reducing bias by correctly specifying relationships (as opposed to multivariable regression where analyses including all variables would control for mediators, for example).

Whereas changing gender attitudes and norms and/or economic resources commonly have been foci for intervention [56], our findings suggest men’s interpersonal violence exposures, trauma symptoms, and gender inequitable attitudes are critical to their use of IPVAW in a conflict-affected setting. If the causal assumptions of our model are correct (e.g., the hypothesized direction between constructs is correct) and to the extent results are generalizable, interventions aiming to reduce IPVAW in conflict-affected settings should adopt new foci on men’s experiences of victimization and mental wellbeing. As experiences of interpersonal violence used in the model were heterogenous regarding timing and setting, however, we recommend additional research to disentangle the impact of violence victimization on IPVAW use, while considering the potential applicability of interventions addressing intergenerational cycles of violence and trauma symptoms. Importantly, these are among the few strategies engaging men that have had positive effects on IPVAW in conflict-affected settings [17, 19].

### Electronic supplementary material

Supplementary table for Tables (PDF 38 kb)

## Data Availability

The data that support the findings of this study now are publicly available.

## Abbreviations

DRC: Democratic Republic of Congo
EFA: Exploratory factor analysis
IPV: Intimate partner violence
IPVAW: Intimate partner violence against women
PTSD: Posttraumatic stress disorder
SEM: Structural equation model
