# Supplementary material for: Drivers of men’s use of intimate partner violence in conflict-affected settings: learnings from the Democratic Republic of Congo
Source: Confl Health. 2024 Jan 22;18:9. doi: 10.1186/s13031-023-00562-5 (PMC10804634; doi:10.1186/s13031-023-00562-5)
Supplement: Supplementary file 1 — Supplementary table for Tables (PDF 38 kb) [file 13031_2023_562_MOESM1_ESM.docx]

Supplement

Figure 1

*Structural equation model of men’s use of IPVAW, controlling for age (n=2080)*


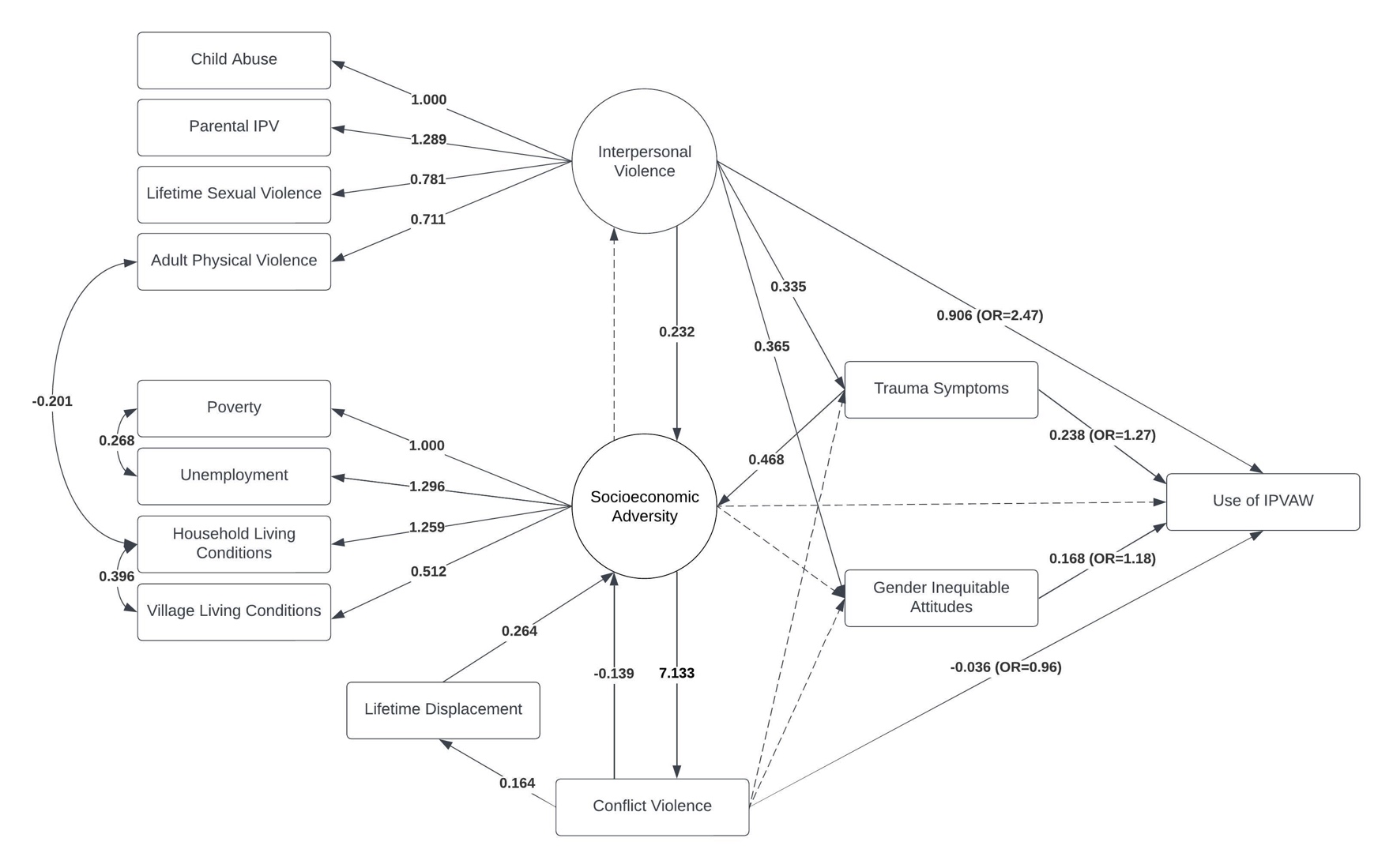


χ^2^=1634.332, p=0.000; RMSEA=0.041; CLI=0.856; SRMR=0.057

Figure 2

*Structural equation model of men’s use of IPVAW, restricted sample (n=1888)*


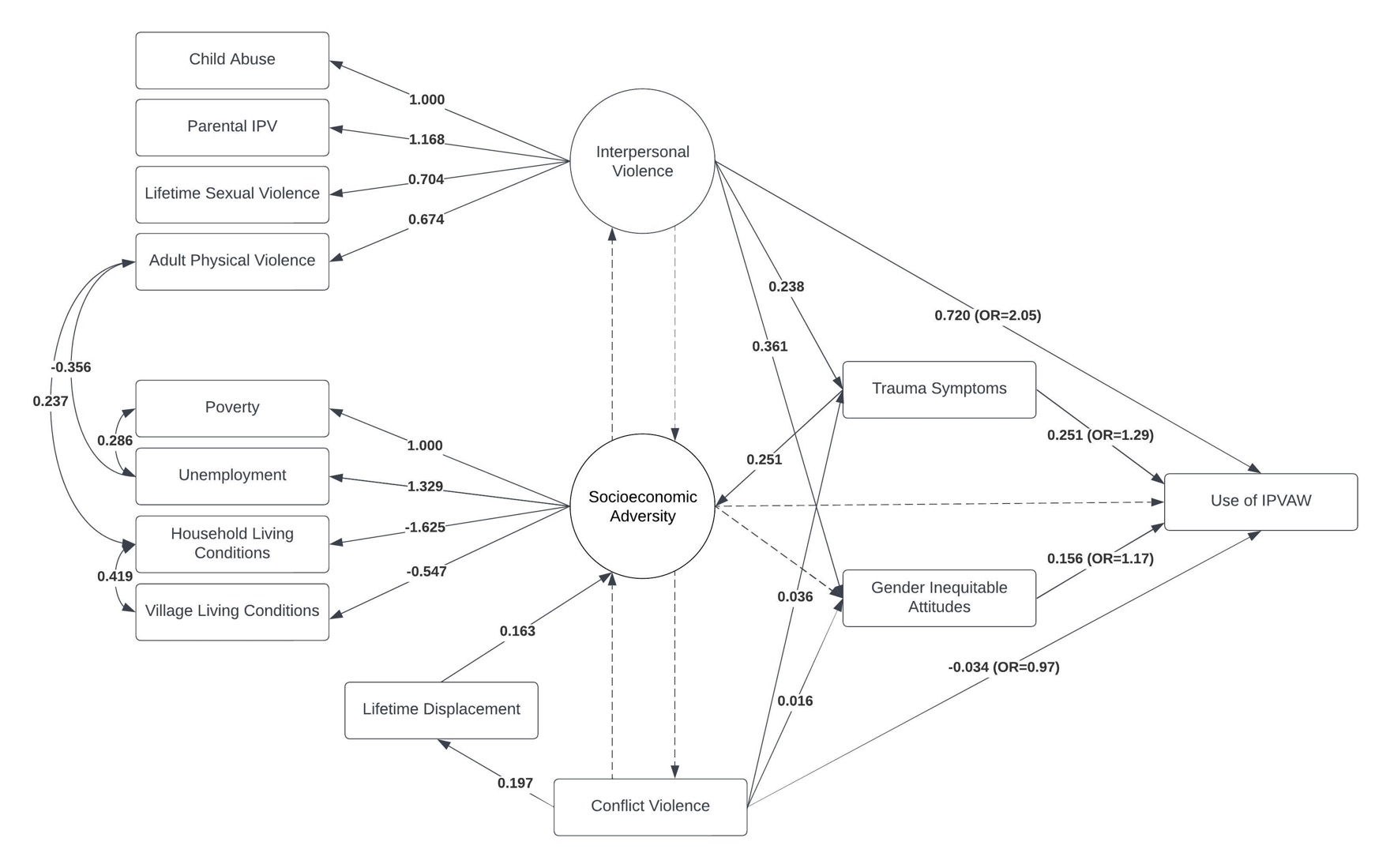


χ^2^= 1458.293, p=0.000; RMSEA=0.049; CLI=0.841; SRMR=0.065

Figure 3

*Structural equation model of men’s use of IPVAW, depression/anxiety (versus trauma) symptoms (n=2080)*


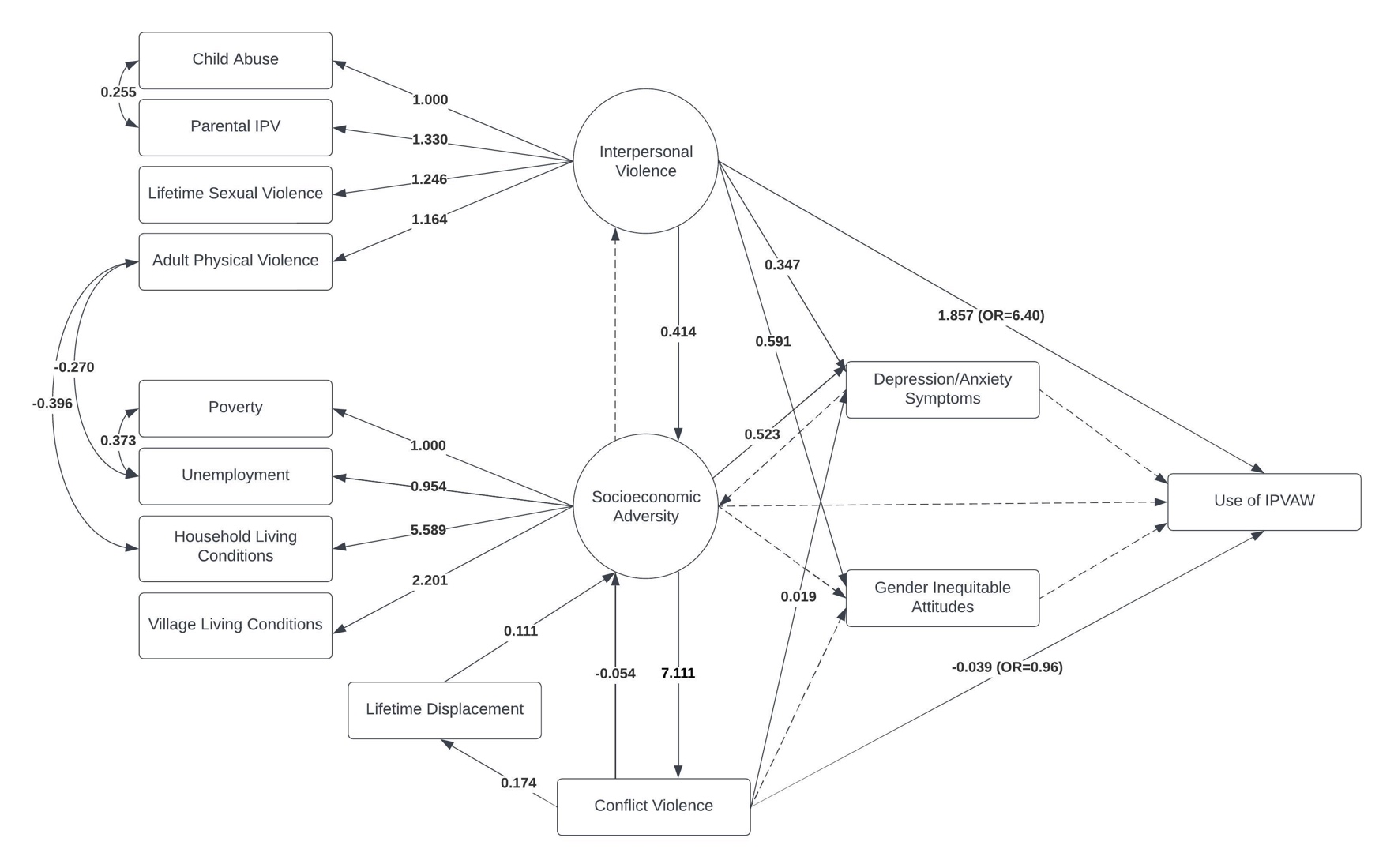


χ^2^= 1659.985, p= 0.000; RMSEA=0.040; CLI=0.896; SRMR=0.052
